# Action Recognition Using a Spatial-Temporal Network for Wild Felines

**DOI:** 10.3390/ani11020485

**Published:** 2021-02-12

**Authors:** Liqi Feng, Yaqin Zhao, Yichao Sun, Wenxuan Zhao, Jiaxi Tang

**Affiliations:** 1 College of Mechanical and Electronic Engineering, Nanjing Forestry University, Nanjing 210037, China; dream6182@163.com (L.F.); kir1160323659@outlook.com (W.Z.); tangjiaxi980413@163.com (J.T.); 2 Kidswant Children Products Co., Ltd., Nanjing 211135, China; yichaosuncn@163.com

**Keywords:** wild feline action recognition, spatial temporal features, two-stream network, deep learning

## Abstract

**Simple Summary:**

Many wild felines are on the verge of extinction, and the monitoring of wildlife diversity is particularly important. Using surveillance videos of wild felines to monitor their behaviors has an auxiliary effect on the protection of wild felines. Through the actions of wild felines, such as standing, galloping, ambling, etc., their behaviors can be inferred and judged. Therefore, research on the action recognition of wild felines is of great significance to wildlife protection. The currently available methods are all aimed at experimental animals and design-specific feature descriptors for specific animals (such as color, texture, shape, edge, etc.), thus lacking flexibility and versatility. The proposed state-of-the-art algorithm using spatial-temporal networks combines skeleton features with outline features to automatically recognize the actions of wild felines. This model will be suitable for researchers of wild felines.

**Abstract:**

Behavior analysis of wild felines has significance for the protection of a grassland ecological environment. Compared with human action recognition, fewer researchers have focused on feline behavior analysis. This paper proposes a novel two-stream architecture that incorporates spatial and temporal networks for wild feline action recognition. The spatial portion outlines the object region extracted by Mask region-based convolutional neural network (R-CNN) and builds a Tiny Visual Geometry Group (VGG) network for static action recognition. Compared with VGG16, the Tiny VGG network can reduce the number of network parameters and avoid overfitting. The temporal part presents a novel skeleton-based action recognition model based on the bending angle fluctuation amplitude of the knee joints in a video clip. Due to its temporal features, the model can effectively distinguish between different upright actions, such as standing, ambling, and galloping, particularly when the felines are occluded by objects such as plants, fallen trees, and so on. The experimental results showed that the proposed two-stream network model can effectively outline the wild feline targets in captured images and can significantly improve the performance of wild feline action recognition due to its spatial and temporal features.

## 1. Introduction

In the past few decades, human activities have caused serious damage to the natural ecological environment, which directly leads to the extinction of a large number of species, including felines. In response, the subject of animal welfare has attracted increasing attention from researchers. Especially, behavioral analysis of wild felines is of great significance to promote animal welfare and helps to enhance the researcher’s understanding of feline habits [1]. The feline population can be more sensitive to public changes, and effective measures can be taken to prevent extinction. The most well-known of the felines are big cats such as lions, tigers, and leopards [2]. Research on their behaviors would promote the development of wild feline welfare [3].

Traditionally, manual annotation of feline activity records is very time-consuming and vulnerable to observers’ prejudice and mental fatigue [4]. Moreover, most of their research objects are confined to a small captive environment [5], which is unable to provide an ideal environment for natural behavior, such as hunting (“hide, track, and chase”) [6,7]. With the development of the Internet of Things technology, the animal information collected by sensors is also used to detect animal behaviors [8,9,10]. However, it may damage the animal’s natural environment and can cause abnormal behaviors to a certain extent [11].

With the rise in computer vision and pattern recognition technology, impressive progress has been made in image classification [12] and object detection [13], motivating researchers to apply artificial intelligence for action recognition of animals, such as mice [14,15,16,17], domestic animals (cows and pigs) [18,19,20,21], Tibetan antelope [22], and ants [23]. However, some of these methods limit the research subjects to small animals in the laboratory [14,15,16,17] while other methods usually make simple judgments about abnormal behaviors of animals [18,19,20,21,22]. Research on wild felines is extremely scarce. Only the work of [24] focuses on tiger behavior. In [24], handicraft characteristics of tigers are extracted and a support vector machine (SVM) is used to classify tiger behaviors.

In order to make up for the absence in feline action recognition, inspired by Pereira et al. [25], who used deep learning to detect Drosophila body parts to analyze its gait patterns, we constructed a two-stream network that incorporates spatial and temporal information for recognizing the action of wild felines, including tigers, lions, and leopards. The spatial element utilizes Mask region-based convolutional neural network (R-CNN) to build an outline detection model and builds a lightweight VGG (Visual Geometry Group) network model by reducing the convolutional layers of VGG16 [26] for action recognition. The temporal part detects the bending angles of the knee joints based on the neural network–based model for animals, called LEAP (Leap Estimate Animal Pose) and recognizes the moving action using LSTM (Long Short-Term Memory) networks.

Our contributions: the contributions of this paper are threefold.

1. We propose a novel two-stream architecture that incorporates spatial and temporal networks for wild feline action recognition. The two-stream network architecture combines the advantages of both the outline features for static action detection and the moving features of the leg skeleton for moving action detection.
2. We build a Tiny VGG network for classifying the outline features extracted by Mask R-CNN. This method can improve the robustness against complex environments due to Mask R-CNN. The Tiny VGG network can also reduce the number of network parameters and avoid overfitting.
3. We present a skeleton-based action recognition model for wild felines. The bending angle fluctuation amplitude of knee joints in a video clip is used as the temporal feature to represent three different upright actions. This model can improve the performance of moving action recognition based on the temporal features, particularly when the animals are occluded by many objects, such as growing plants, fallen trees, and so on.

Organization: the rest of the paper is organized in the following way. Section 2 describes recent studies related to our work. Section 3 presents a two-stream network, including an outline-based spatial stream and a skeleton-based temporal stream, for wild feline posture recognition. The experimental results are discussed in Section 4, while the conclusions are drawn in Section 5.

## 2. Related Work

Deep learning algorithms have been widely applied to image classification and human behavior recognition due to their excellent performance and abilities that are suitable for large-scale learning [27,28,29,30]. Some researchers have built deep convolutional neural network models for species identification [31,32,33,34,35,36]. Gómez et al. [31] constructed a deep convolutional neural network (DNN) for classifying wild animal images. However, these methods require substantial human effort. To fill the gap between field image data acquisition and data analysis, the automatic image analysis system, ClassifyMe, was designed to automatically identify animal species [32]. In [31], the experimental images were manually cropped, and then, those cropped patches containing the animals were selected. Chen et al. [33] tried to automatically crop animals from images and to classify wild animal species based on a deep convolutional neural network but only reached an accuracy of 38%, thus leaving much room for improvement. To improve the classifying performance for wild animal species, Norouzzadeh et al. [34] trained nine different DNN models and formed an ensemble of the trained models by averaging their predictions. Although the accuracy was increased to more than 95% in the Snapshot Serengeti dataset, training nine DNN models is clearly computationally expensive.

Besides classifying wild animal species, a few researchers have focused on harnessing machine learning algorithms to identify the behaviors of wild animals. In [21], two binary-classifier support vector machines were performed in a hierarchical manner, but they could only make simple judgments, such as whether the pigs have aggressive behaviors. Luo et al. [22] analyzed the behavior of Tibetan antelope (panthoops hodgsonii) in the Tibetan Plateau and was the first to call the behavior standing for a certain time as “puppet resting behavior”, which is an adaptive form of rest. Norouzzadeh et al. [34] attempted to identify the species, to count the animals, to describe the animal behaviors, and to determine the presence of young. Zhang et al. [35] used Omni-supervised joint pose estimation and detection for kangaroos in a dataset that was collected in several national parks across Queensland State during 2013 in Australia. The ZooMonitor application (app) was designed by Lincoln Park Zoo to monitor the behavior, habitat use, and appearance of animals in a low-cost, flexible manner [36]. Considering the difficulty in acquiring animal behavior images, Zuffi et al. [37] tried to build a statistical shape model of the 3D poses and shapes of animals and then fitted this model to 2D image data. The experimental dataset was developed from 3D scans of toy figurines in arbitrary poses. Bod’ová et al. [22] proposed a probabilistic model of animal behavior that combined deterministic dynamics and stochastic switching between these states. In [23], two interacting ants were chosen as an example to capture a wide variety of complex individual and collective behaviors. However, the performance of these two models needs to be further validated for behavior analysis and pose detection of wild animals living in uncontrolled natural environments. Zhang et al. [35] mainly focused on multi-class wildlife detection in an Omni-supervised learning setting. In [35], the pose estimation for kangaroos was also briefly introduced, but the types of poses only included scenes where the body parts faced the camera. In a strict sense, poses do not belong to the behaviors of wild animals and are barely related to actual behaviors, such as standing, galloping, ambling, etc.

The most closely related work is [23]. To our best knowledge, this is the only attempt to identify the actions of wild animals using real camera-trap images. In [36], some common actions, such as standing and resting, were identified by an ensemble model that averaged the prediction results of nine different trained DNN models. Obviously, this model not only requires costly hardware configurations but also is computationally expensive due to the need to train nine different DNN models. Furthermore, this model also cannot recognize moving actions such as ambling and galloping.

## 3. Methods and Materials

**Figure 1:**
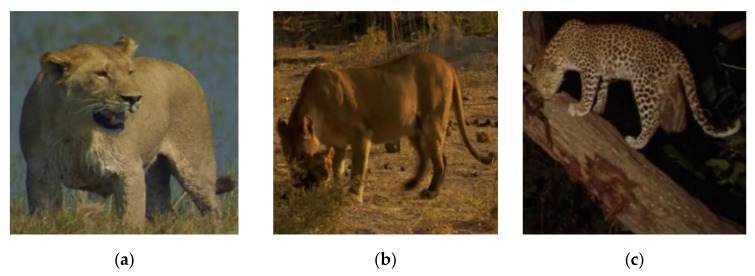
Examples of challenging images: (**a**) an image occluded by other objects, (**b**) an image camouflaged by the surroundings, and (**c**) an image taken at night.

Unlike most object detection tasks, wild feline detection has markedly more difficult challenges due to illumination changes and a complex background [38]. Figure 1 provides some examples of such challenging images. The felines can be occluded by many objects such as growing plants, fallen trees, etc. (see Figure 1a). In autumn, the color of feline hair is similar to that of withered grass or leaves, so the animals can be camouflaged by the surroundings (see Figure 1b). It is, therefore, challenging to detect the existence of felines in a dark environment at night, as Figure 1c shows.

### 3.1. Pipeline Overview

**Figure 2:**
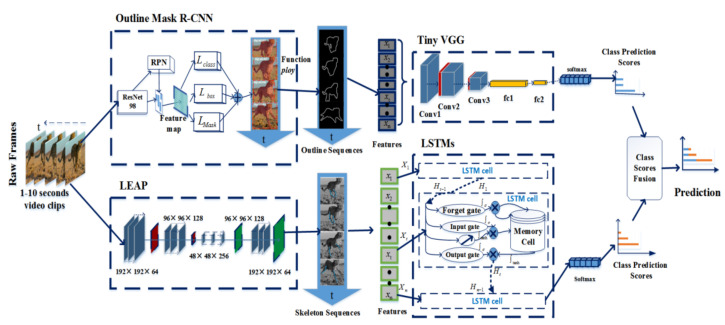
Workflow of the wild feline action recognition.

The pipeline of our framework is depicted in Figure 2. The framework is a two-stream architecture that incorporates spatial and temporal networks for wild feline action recognition. In the spatial part, the wild felines are outlined on the basis of the masks generated by Mask R-CNN (this method is called Outline Mask R-CNN). Then, the outlines are imported into a lightweight VGG network (named Tiny VGG) to learn the outline features. In the temporal part, the skeleton features in an animal video clip are extracted by tracking the animal body parts based on LEAP (Leap Estimate Animal Pose). After that, the LSTMs are used to learn the temporal features in the skeleton sequences. Finally, the weighted average operation is used as a fusion strategy to combine the predictions from both streams.

### 3.2. Construction of the Outline Model

#### 3.2.1. Outline Mask RCNN

Mask R-CNN [39] predicts segmentation masks on each Region of Interest (RoI), in parallel with the branch for classification and bounding box regression. A small FCN (Fully Convolutional Network) [40,41] is applied to each RoI to predict the segmented mask in a pixel-to-pixel manner. With its excellent performance, Mask R-CNN is popular in object detection, instance segmentation, and key-point detection tasks [42,43,44]. In this paper, we build a feline object detection model based on Mask R-CNN and then extract the object outline information.

Here, we use transfer learning to reduce the need for a large number of data and to improve the generalization ability of the model. The training data for transfer learning come from four-legged mammal images that contain the whole animal body in the Snapshot Serengeti dataset [45] and the COCO database [46]. Before transfer learning, the object outlines of the training images must be marked to realize feline segmentation.

In general, we can obtain richer feline characteristics using a deeper network structure. However, the network can degenerate with the deepening of the network due to gradient dispersion. The deep residual network ResNet [47] adds identity mapping for skip connection, which can avoid the gradient dispersion of a deep network structure. Therefore, we use ResNet here to construct an Outline Mask Region-based Convolutional Neural Network (Outline Mask R-CNN) for wild feline images.

During training, we defined the multitask loss on each sampling RoI using Equation (1):

(1) $$\begin{array}{l} loss=mrcnn\_bbox\_loss+mrcnn\_class\_loss+mrcnn\_mask\_loss+\\ rpn\_bbox\_loss+rpn\_class\_loss \end{array}$$

where the functions mrcnn_bbox_loss, mrcnn_class_loss, mrcnn_mask_loss, rpn_bbox_loss, and rpn_class_loss represent mask edge regression loss, mask classification loss, mask average binary cross-entropy loss, regional recommendation regression loss, and regional recommendation classification loss, respectively. These loss functions are identical to those defined in [39].

In the proposed method, the objects recognized are only the felines, and other objects such as trees, flowers, and grass are treated as background. Therefore, a very deep network is not a reasonable choice. Thus, we simplified the layers of the ResNets and adjusted the network parameters continuously to build a feline object detection model based on Mask R-CNN. The numbers of layers of the simplified ResNets changed from 50 to 101. The parameters of the three simplified ResNets are shown in Table 1.

**Figure 3:**
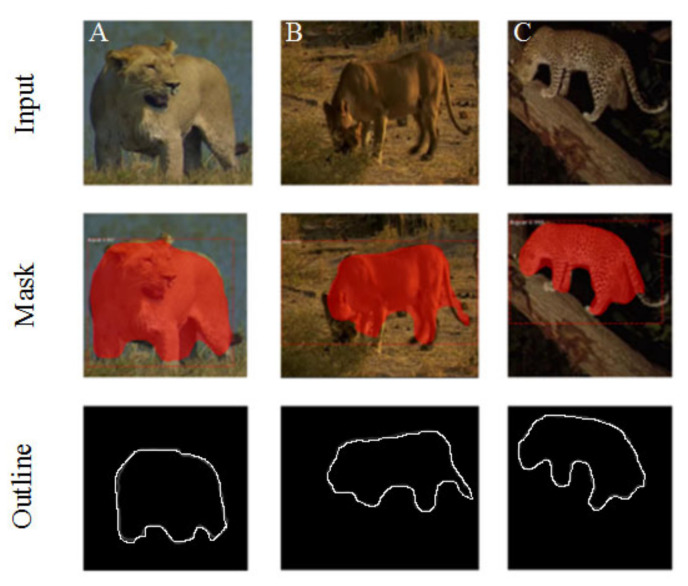
Example results of the Outline Mask region-based convolutional neural network (R-CNN) (**Top**: original images of felines in different poses; **middle**: masks extracted by Mask R-CNN; and **bottom**: outline extracted by Outline Mask R-CNN).

Afterwards, we used the function “ploy” in the Matplotlib image library to extract the outlines of the masks generated by Mask R-CNN. As shown in Figure 3, Outline Mask R-CNN can effectively detect the object outlines of wild feline images captured in some complex field environments (such as occluded felines (e.g., Figure 3A), camouflaged felines (e.g., Figure 3B), and animal images taken at night (e.g., Figure 3C).

#### 3.2.2. Tiny VGG for Action Classification

**Figure 4:**
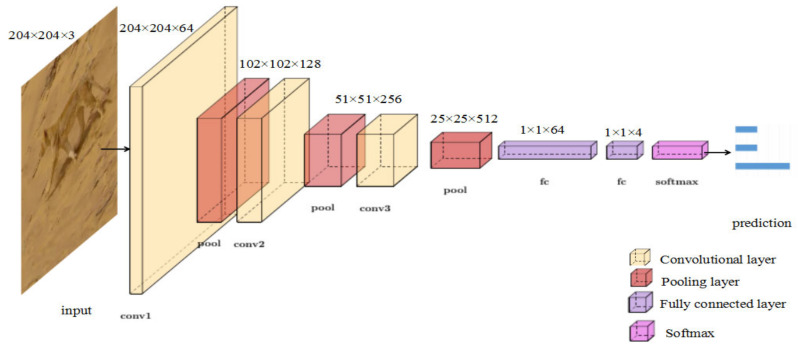
Illustration of Tiny Visual Geometry Group (VGG).

VGG (Visual Geometry Group) was proposed by Oxford Visual Geometry Group in 2015, which investigated the efforts of a convolutional network depth on its accuracy in a large-scale image recognition setting. VGG can help to learn more robust features from graph structure data. Rather than using relatively large size convolution kernels [48], the VGG uses very small 3×3 convolution kernels throughout the whole network. In view of this, a lightweight convolutional neural network Tiny VGG was constructed to reduce the number of network parameters and to avoid overfitting when classifying the different actions of wild animals. As shown in Figure 4, similar to the network structure of VGG, the parameters of Tiny VGG are provided in Table 2. To obtain the final prediction, we connected two fully-connected layers after the last convolutional layer to map the extracted features to the categories. Then, we ran a SoftMax [48] operation, which is widely used in classification tasks, on the output to obtain the predicted probabilities. The predicted probability p of output y for the nth class given a sample vector x and a weighting vector w is defined using Equation (2):

(2) $$p\left(y=n|x\right)=\frac{e^{x^{T}w_{n}}}{\sum_{k=1}^{K}e^{x^{T}w_{k}}},$$

where $x^{T}w\;$denotes the inner product of x and w. This can be seen as the composition of K linear functions.

### 3.3. The Construction of the Skeleton Model

#### 3.3.1. Tracking the Position of the Animal’s Leg Joints

**Figure 5:**
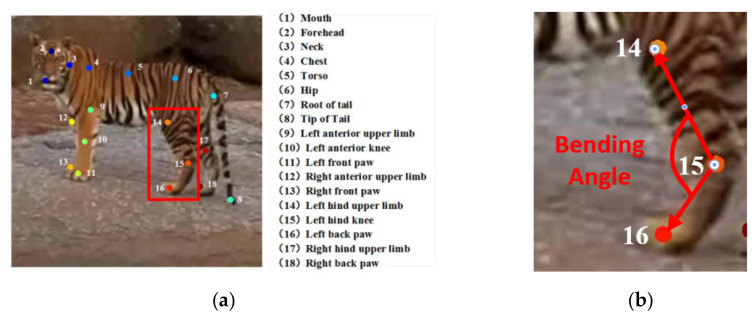
Joint point-labeled skeleton: (**a**) the eighteen tracked points; (**b**) the bending degrees of the knee joints (zoom in).

Just as human action is mainly determined by limbs, animals’ actions, such as standing, ambling, and galloping, are mainly determined by leg movement. Therefore, we used LEAP, which is an automated and efficient system consisting of a Graphical User Interface (GUI)-driven workflow for labeling images and a deep-learning-based network for predicting the positions of animal body parts [23] to track the positions of the animal’s leg joints. The key frames in the wild feline video clip were extracted by k-means clustering [51]. The joint positions of the key frames were labeled and used to train the LEAP network. After that, the LEAP network generated body-part estimates for the remaining images in the video clip. We also used these estimates as the initial values in the GUI (Graphical User Interface) to predict a new video clip. As shown in Figure 5a, we tracked 18 distinct points to describe the poses of the head, body, tail, and legs and chose 6 points of two uncovered legs on the outer side to express the status of leg movement. The bending degree of the knee joints is shown in Figure 5b.

#### 3.3.2. Action Identification Based on Skeleton

As shown in Figure 5b, taking the tiger’s hind limb as an example, the knee joint (denoted as “15” in Figure 5b) is the vertex of the bending angle, and the smaller included angle between the femur and tibia is defined as the bending angle of one knee joint. The bending degrees of the knee joints obviously vary according to the animal posture. When the longitudinal axis of the feline is parallel to the screen plane, the bending angle is computed using Equation (3):

(3) $$\begin{array}{l} L_{12}=\sqrt{(x_{14}-x_{15}{)}^{2}+{\left(y_{14}-y_{15}\right)}^{2}}\\ L_{13}=\sqrt{(x_{14}-x_{16}{)}^{2}+{\left(y_{14}-y_{16}\right)}^{2}}\\ L_{23}=\sqrt{(x_{16}-x_{15}{)}^{2}+{\left(y_{16}-y_{15}\right)}^{2}}\\ Bending\_Angle=\arccos(\frac{L_{12}{}^{2}+L_{23}{}^{2}-L_{13}{}^{2}}{2\times L_{12}\times L_{23}}) \end{array}$$

where $x_{k}$ and $y_{k}$ are the coordinates of the kth key-point.

**Figure 6:**
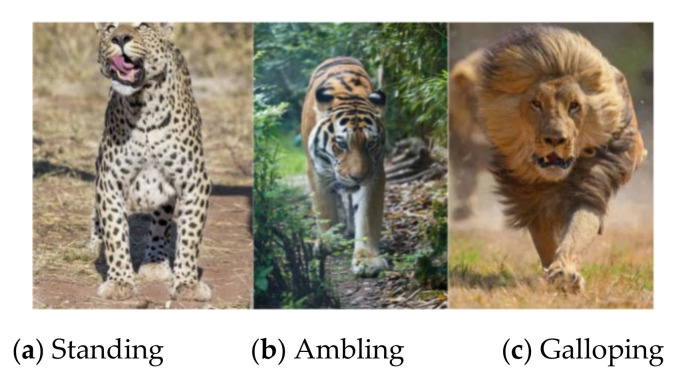
Images in which felines’ spines are perpendicular to the image plane in space: from this perspective, the angle of leg joints is almost straight regardless of species and the states of felines.

However, when the longitudinal axis of the feline is not parallel to the screen plane, there will be a certain error when calculating the bending angle using Equation (3). In this case, the closer the angle between the feline’s spine direction and the screen plane is to 0°, the more accurate the calculation of the bending angle is; on the contrary, the closer the angle between the feline’s spine direction and the plane is to 90°, the greater the deviation of the bending angle calculation is. Until the feline’s spine is spatially perpendicular to the plane, no matter what movement state the animal is in, the joints of the feline will form a straight line (Figure 6). In this situation, it is unable to distinguish different movement states by the bending angle change.

**Figure 7:**
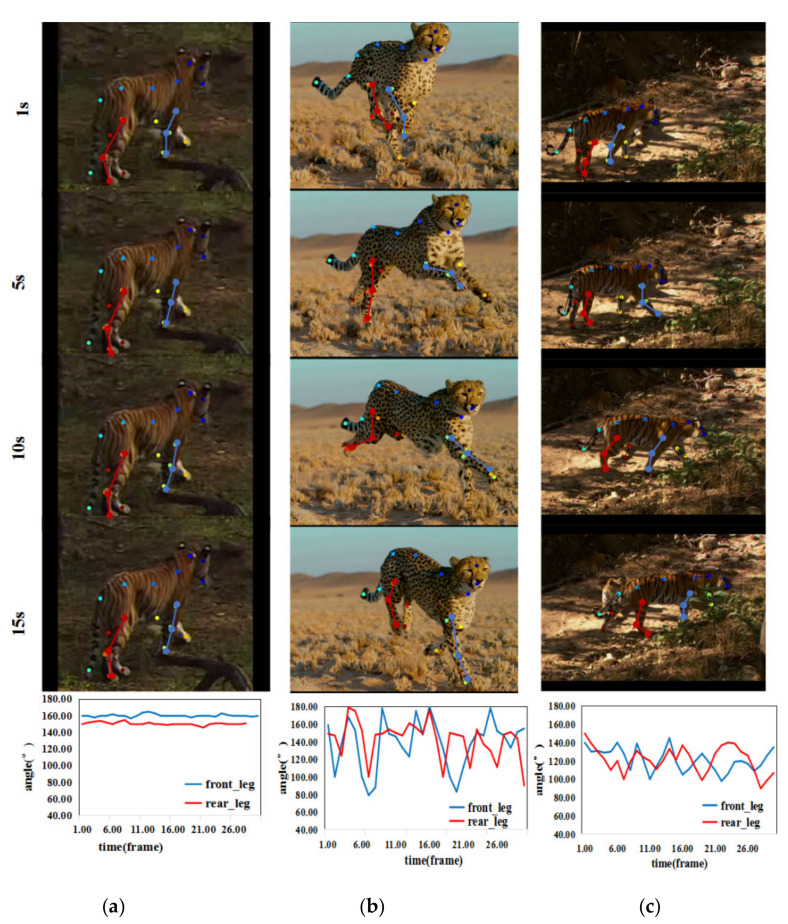
Video representative frames and variation in the bending angle: from the first row to the fourth row are video representative frames of three upright actions and the fifth row is the variation curves of the bending angles (blue and red represent the variation curves of the front legs and hind legs, respectively) for (**a**) standing, (**b**) galloping, and (**c**) ambling.

Under the circumstance that the animal’s spine direction is at an angle $\left(0{}^{\circ},90{}^{\circ}\right)$ with respect to the screen plane, there is a certain deviation between the bending angle and the true angle calculated by Equation (3). However, the change of bending angle is still very representative. Figure 7 shows the variation in the bending angle in the three video clips representing three types of different upright actions. The bending angle has the largest fluctuation amplitude during animal galloping. It fluctuates between approximately 80° and 180°, while the bending angle of the ambling posture fluctuates less. The bending angle of the standing posture has the smallest fluctuation amplitude, which ranges from approximately 140° to 160°. Hence, we can draw the conclusion that, over time, the angles of leg joints vary obviously under different movement states of animals.

We computed the variation in the bending angles in a video sequence instead of a single image or adjacent frame. LSTM [52] is a variant of RNN (Recurrent Neural Networks) [53], which contains multiple LSTM cells. Each cell follows the ingenious gating mechanism (first, the forget gate decides what to discard in the previous cell state; then, the input gate updates information; and finally, the output gate transmits filtered information to the next cell state), which makes LSTMs capable of learning long-term dependencies. Therefore, we used LSTMs to recognize the different actions of the animals according to the sequence of the bending angles in the video clip.

### 3.4. Score Fusion

To exploit the complementation between outline-based Tiny VGG and skeleton-based LSTMs, we next took the weighted average as the fusion strategy and obtained the final prediction. Let $y_{p}$ and $y_{s}$ denote the scores of the outline and skeleton stream, respectively. The final prediction y is defined as Equation (4):

(4) $$y=\partial \cdot y_{p}+\left(1-\partial \right)\cdot y_{s},$$

where ∂ is the relative weight of the two stream predictions and the range for $y_{s},\;y_{p},\;y$ and ∂ is (0, 1).

### 3.5. Materials

#### 3.5.1. Configurations

For machine learning, the larger the sample size and the more images the machine learns, the more accurately the network can recognize different actions of wild felines. For this research, the machine needs very powerful processing and computing power. Therefore, we set up a TensorFlow-GPU environment in our local personal computer (PC) to conduct all experiments. Additionally, the environment has an NVIDIA GEFORCE RTX 2080Ti graphics card that supports TensorFlow-GPU version 1.6.0 and Windows 10 operating system for image processing. CUDA version 10 provides additional processing power for the computer to use the video card when training the model. Additional commercial or third party software were also used: MathWorks MATLAB R2018a and Python 3.6.4. The required libraries were installed via the pip package manager: numpy (v.1.14.1), h5py (v.2.7.1), tensorflow-gpu (v.1.6.0), keras (v.2.1.4), scipy, pillow, cython, matplotlib, scikit-image, opencv-python, and imgaug.

#### 3.5.2. Data Collection

In this paper, we recognized wild feline action by using video sequences. At present, all the public datasets released for researches on wildlife recognition are on the basis of the images of wildlife. It is not easy to record videos of wild felines because of the shy characteristics of wild felines and the restrictions to their living environment. Therefore, video datasets for wild felines are scarce, and there is no public video dataset available for this particular task. Hence, by using a Python script adapted from a web scraping tool created by Hardik Vasa [54], 90 full high-definition (HD) resolution documentaries were collected from http://www.05jl.com and other websites about wild felines. In a similar process, Kody G. Dantongdee of California State University San Luis Obispo also used a similar process to complete the image collection work for his project [55]. These documentaries can help to accurately reflect the living conditions of wild felines. Based on the criteria that the animal movement patterns can be clearly observed from a side perspective, we manually intercepted meaningful video clips from documentaries about wild felines. Each video clip at 30 frames per second (FPS) contains the process of performing a single action of the target feline.

#### 3.5.3. Data Preprocessing

**Figure 8:**
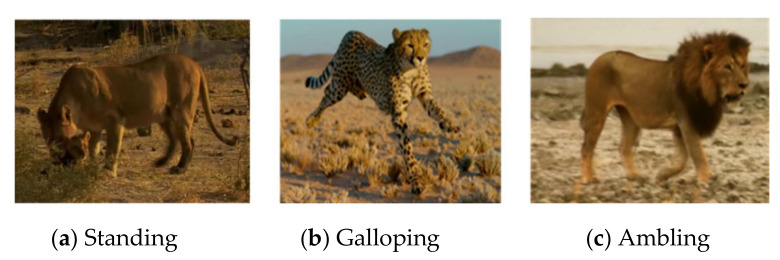
Example of the dataset.

The dataset contains three types of felines, namely tigers, lions, and leopards. The experimental data were segmented into about 2700 small video clips lasting less than ten seconds, with three different actions (standing, galloping, and ambling) labeled, as shown in Figure 8. Each action dataset contained about 900 video clips, 500 of which were randomly selected for training, with the remaining 400 video clips used for testing. To make learning easier for the neural networks, data processing is necessary. For LEAP, to ensure consistency in output image size after repeated pooling and upsampling in the neural network of LEAP, we followed standard practices in scaling down the images to 192 × 192 pixels. Then, we converted all video clips to HFD5 files using the Python code and used self-describing HDF5 files as input to train the network. For Outline Mask RCNN, we converted video clips into continuous frame images using the Python code. Then, the images in each video clip were resized to 112 × 112 for instance segmentation.

## 4. Results and Analysis

### 4.1. Outline Classification-Based Action Recognition

#### 4.1.1. Outline Mask R-CNN

The model’s backbone, ResNet, was initialized with the publicly released weight pre-trained on the COCO dataset and then fine-tuned with our object box annotations. All the pixels in one image were divided into only two categories: the background type and the target feline involved in the posture. Twenty epochs were used in the training process, and each epoch was iterated 100 times.

**Figure 9:**
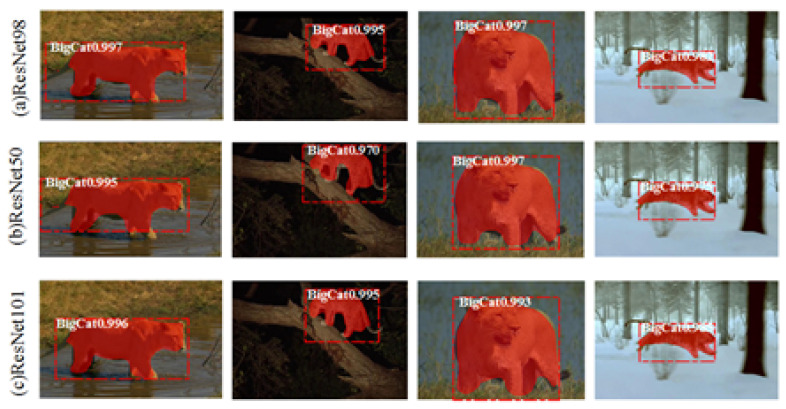
Instance segmentation results for four different actions (first row: ResNet98, second row: ResNet50, and last row: ResNet101; from left to right: ambling, two different types of standing, and galloping).

**Figure 10:**
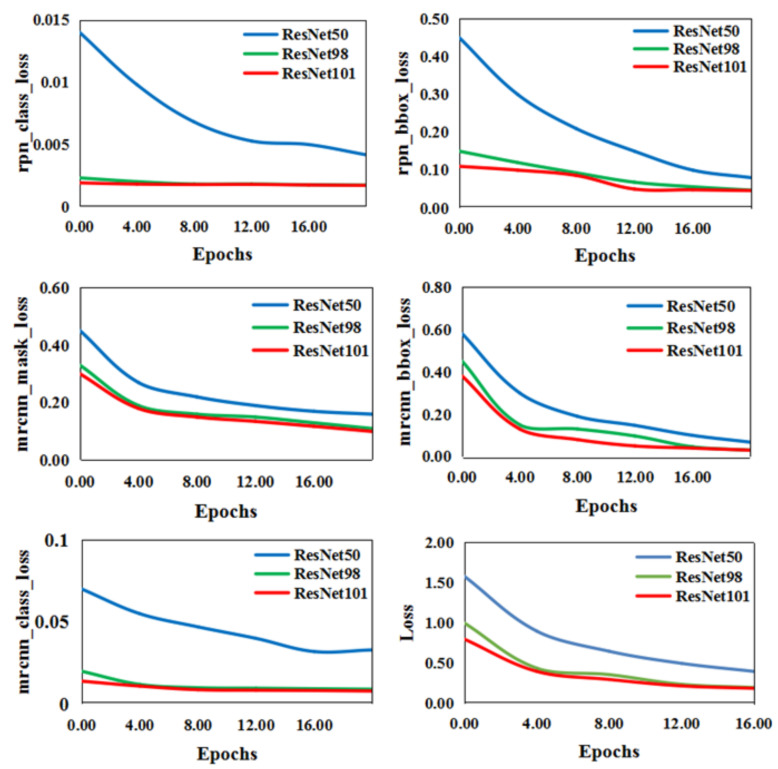
The convergence curves of the six loss functions with different numbers of layers. (ResNet50, ResNet98, and ResNet101 represent the number of layers being 50, 98, and 101, respectively. The blue, green, and red lines correspond to convergence of the loss function when the number of ResNet network layers is 50, 98, and 101, respectively.)

As mentioned in Section 3.2.1, the number of the layers in simplified ResNets changed from 50 to 101. Figure 9 shows that, even in unfavorable external environments (nights and heavy snow) or terrain obstacles (weeds and river water) where part of the wild feline’s legs were covered, the animal regions extracted were comparatively complete, regardless of whether the layers were 50, 98, or 101 (named ResNet50, ResNet98, and ResNet101, respectively). However, detailed information, such as tails, could not be detected, and two adjacent limbs sometimes could not be separated. The five loss functions mentioned in Section 3.2.1 were used for convergence of the three different ResNet-size networks. As shown in Figure 10, the convergence of ResNet50 was substantially slower than those of ResNet101 and ResNet98. Meanwhile, the test time of ResNet98 (0.57s per image) was less than that of ResNet101 (0.59s per image). According to the experimental results, the number of layers was set to 98 by considering the balance between the convergence and time-consuming of ResNet-size networks.

#### 4.1.2. Tiny VGG for Action Classification

This model was optimized by the Adam optimization algorithm [56], which dynamically adjusts the learning rate of each parameter to make the parameters change steadily, and the learning rate was set to 0.01. The cross-entropy loss was selected as the loss function for the recognition task. Thirty epochs were used in the training process with 100 iterations per epoch for a total of 3000 iterations.

In image classification, VGG16, Mobile Net V2 [57], and Inception V3 [58] are three classic deep learning networks, but if these networks are trained directly on our data, it is likely to cause problems such as network degradation and overfitting. Thus, by simplifying the model to adapt to this task, we constructed three tiny convolutional networks based on Inception V3, MobileNet V2, and VGG, respectively. Tiny Inception V3 applies three convolution kernels with different scales (1 × 1, 3 × 3, and 5 × 5) to train the network, and the parameters of simplified Inception V3 are shown in Table 3. Tiny MobileNet V2 is a lightweight network structure based on depthwise separable convolution, and the parameters of simplified MobileNet V2 are shown in Table 4.

**Figure 11:**
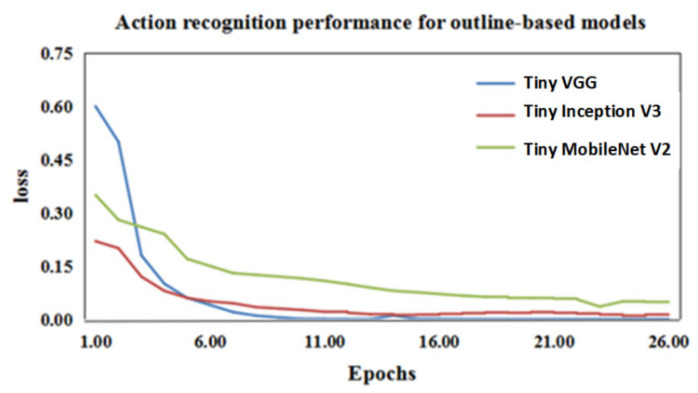
Convergence curve of the loss function for the three tiny convolutional networks: the blue, red, and green lines represent the convergence of the loss functions of the recognition networks Tiny VGG, Tiny Inception V3, and Tiny MobileNet V2, respectively.

As shown in Figure 11, the convergence rate of Tiny VGG is faster than that of the other two models. Table 5 shows the classifying accuracy of the three convolutional networks. The mean accuracy of Tiny VGG reached 92%, which is 10% higher than Tiny MobileNet V2. Therefore, Tiny VGG was used to recognize the three actions of the wild felines according to the outline feature extracted by Outline Mask R-CNN.

### 4.2. Skeleton Classification-Based Action Recognition

**Figure 12:**
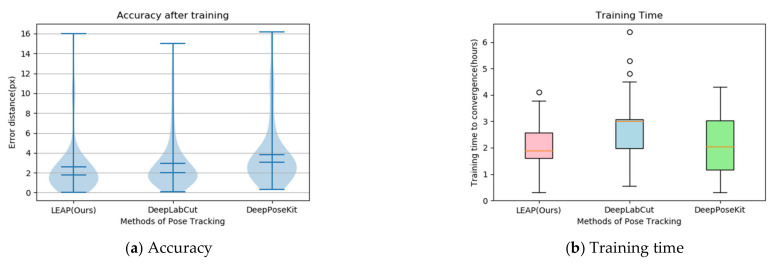
Accuracy and training time for the three models tracking joint points: (**a**) violin plots denote the overall error distribution, and the error bars denote the 25th and 75th percentiles; (**b**) the red, blue, and green boxes plot the training times of LEAP (Leap Estimate Animal Pose), DeepLabCut, and DeepPoseKit, respectively.

We carried out comparative experiments on tracking joint points to determine the tradeoffs between speed and accuracy in the three models (the LEAP model, DeepLabCut model [59], and DeepPoseKit model [60]). Figure 12a shows the error distribution and accuracy. The violin plot is good at expressing the distribution status and probability density between different categorical variables. The wider the area, the more data distributed [61]. The overall error distribution of LEAP is almost equal to that of DeepLabCut but less than that of DeepPoseKit. The accuracy of LEAP is superior to that of DeepLabCut and almost equal to that of DeepPoseKit. Figure 12b reveals the training time to convergence. The box plot compares the distribution characteristics of different networks in the training time, which mainly contains 6 key nodes: the data, the upper edge, the upper quartile Q3, the median, the lower quartile Q1, and the lower edge. When a data point is outside the range of $\left[Q_{1}-1.5IQR,Q3+1.5IQR\right]$ (where $IQR=Q_{3}-Q_{1}$), it is considered an outlier [62]. The time needed for training LEAP is shorter than that of the two other models. Thus, the LEAP model was used to track the joint points of wild felines.

The Adam optimization algorithm was used to optimize the model. The initial learning rate was set to 0.001 and reduced by a factor of 10 until the validation loss was no longer reduced. The mean squared error between the predicted and ground-truth map was selected as the loss function for skeleton estimation.

### 4.3. Our Two-Stream Model

**Figure 13:**
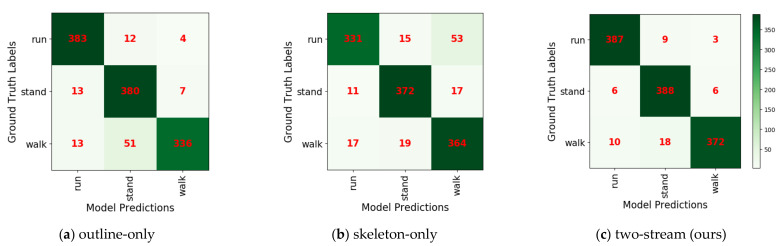
Confusion matrix for the three proposed models: all correct predictions are located on the diagonal of the matrix; it is easy to visually inspect the number of feline actions with incorrect predictions, as they are represented by values outside the diagonal.

Because of the lack of research on wild feline posture classification, we can only compare our two-stream model with the two single-stream models proposed in this paper. The outline-only model sometimes failed to distinguish ambling actions from standing actions because the outline features of ambling are similar to those of standing, particularly when the two limbs are adjacent. As shown in Table 6, the outline-only model presents a strong performance for galloping (96%) but fails with ambling (84%). The skeleton-only model can accurately distinguish ambling and standing due to temporal features. However, the variation of the bending angle is not always obvious for some galloping actions, which may be confused with ambling. As shown in Table 6, the skeleton-only model achieved a good performance for ambling (91%) but underperformed for galloping (83%). After fusion, the proposed two-stream method provided robust performance in recognizing the three actions. Figure 13 shows the confusion matrixes of the three actions classified by the outline-only model, skeleton-only model, and our two-stream model. The outline-only model mistook 12.75% of the ambling samples for standing. In the skeleton-only model, 13.25% of the galloping samples were incorrectly classified as ambling. The two-stream model is thus superior to the two single-stream models.

## 5. Conclusions and Future Work

This paper is the first to present an action recognition method for wild felines. This method can effectively outline the wild animal region and can recognize standing and galloping actions. However, this method sometimes fails to distinguish between ambling and standing, particularly when the two limbs are adjacent. Therefore, a novel skeleton-based action recognition model is established to use the bending angle fluctuation amplitude of knee joints as temporal features. The model offers superior performance in classifying ambling and standing, but it sometimes incorrectly identifies galloping as ambling when the variation of the knee bending angle is not obvious. To take advantage of both, this paper proposed a novel two-stream architecture that incorporates spatial and temporal networks. The proposed two-stream network model can significantly improve the performance of wild feline action recognition based on spatial and temporal features.

Although we successfully applied a two-stream network for feline action recognition, there is still a lot of improvement to be made. In the future, we will collect more gait videos, such as trot, pace, canter videos, etc., to delve into the behavior of wild felines. Additionally, events such as unfavorable weather conditions (fog, rain, etc.) or the presence of tall terrain obstacles that occlude the whole legs or most of the body will be considered to make the research more comprehensive. Theoretically, this method is suitable for action recognition of other four-legged mammals. We will use this algorithm in the assessment of other animal species, e.g., Cervidae in Europe or small mammals. In order to improve the robustness of the system, video noise and other artifacts affecting the quality of the images will be further added to train the network. Meanwhile, inspired by two-person interaction action recognition [63,64], we will try to apply the GNN (graph neural network) algorithm [65] to the recognition research of multi-animal interaction behavior by transfer learning. Furthermore, an attention mechanism [66] will be introduced to pay more attention to the changes in joints and bones caused by animal movement, not only changes in leg joint angle, so that animal moving images taken from all angles can be accurately identified by the network. Therefore, we will design a more robust, comprehensive network for estimating wildlife action recognition in the future.

## Figures and Tables

**Table 1:** The structures of the ResNets: ResNet50, ResNet98, and ResNet101 represent that the number of layers is 50, 98, and 101, respectively.

Layer	Output Size	ResNet50	ResNet101	ResNet98
Conv_1	112×112	$\left[\begin{array}{cc} 7\times 7 & 64 \end{array}\right]$		
Conv_2	28×28	$\left[\begin{array}{cc} 1\times 1 & 64\\ 3\times 3 & 64\\ 1\times 1 & 256 \end{array}\right]\times 3$	$\left[\begin{array}{cc} 1\times 1 & 64\\ 3\times 3 & 64\\ 1\times 1 & 256 \end{array}\right]\times 3$	$\left[\begin{array}{cc} 1\times 1 & 64\\ 3\times 3 & 64\\ 1\times 1 & 256 \end{array}\right]\times 3$
Conv_3	14×14	$\left[\begin{array}{cc} 1\times 1 & 128\\ 3\times 3 & 128\\ 1\times 1 & 512 \end{array}\right]\times 4$	$\left[\begin{array}{cc} 1\times 1 & 128\\ 3\times 3 & 128\\ 1\times 1 & 512 \end{array}\right]\times 4$	$\left[\begin{array}{cc} 1\times 1 & 128\\ 3\times 3 & 128\\ 1\times 1 & 512 \end{array}\right]\times 3$
Conv_4	7×7	$\left[\begin{array}{cc} 1\times 1 & 256\\ 3\times 3 & 256\\ 1\times 1 & 1024 \end{array}\right]\times 6$	$\left[\begin{array}{cc} 1\times 1 & 256\\ 3\times 3 & 256\\ 1\times 1 & 1024 \end{array}\right]\times 23$	$\left[\begin{array}{cc} 1\times 1 & 256\\ 3\times 3 & 256\\ 1\times 1 & 1024 \end{array}\right]\times 22$
Conv_5	7×7	$\left[\begin{array}{cc} 1\times 1 & 512\\ 3\times 3 & 512\\ 1\times 1 & 2048 \end{array}\right]\times 3$	$\left[\begin{array}{cc} 1\times 1 & 512\\ 3\times 3 & 512\\ 1\times 1 & 2048 \end{array}\right]\times 3$	$\left[\begin{array}{cc} 1\times 1 & 512\\ 3\times 3 & 512\\ 1\times 1 & 2048 \end{array}\right]\times 3$
Average Pooling	1×1	1000 dimensions		

**Table 2:** The structures of the ResNets: the convolutional layer parameters are denoted as “Conv (receptive field size [49]) (number of channels)”; the ReLU [50] activation function is not shown for brevity.

Layer	Patch Size	Stride
Conv1_64	3 × 3	1
Max Pooling	—	2
Conv3_128	3 × 3	1
Max Pooling	2 × 2	2
Conv3_256	3 × 3	1
Max Pooling	2 × 2	2

**Table 3:** Structure of Tiny Inception V3: the building blocks are shown, with the size of the filter bank and the numbers of stride stacked.

Layer	Filter Shape/Stride
Conv2d_bn	1 × 1 × 32/1
Conv2d_bn	3 × 3 × 32/1
Conv2d_bn	3 × 3 × 64/2
Max Pooling	Pool 3 × 3/2
Conv2d_bn_1_1	1 × 1 × 64/1
Conv2d_bn_1_5	1 × 1 × 48/1
Conv2d_bn_1_5	5 × 5 × 64/1
Conv2d_bn_1_3	1 × 1 × 64/1
Conv2d_bn_1_3	3 × 3 × 96/1
Average Pooling	Pool 1 × 1/1
Conv2d_bn_Pool	1 × 1 × 32/1
Conv2d_bn_2_1	1 × 1 × 64/1
Conv2d_bn_2_5	1 × 1 × 48/1
Conv2d_bn_2_5	5 × 5 × 64/1
Conv2d_bn_2_3	1 × 1 × 64/1
Conv2d_bn_2_3	3 × 3 × 96/1
Conv2d_bn_2_3	3 × 3 × 96/1
Average Pooling	-
Conv2d_bn_Pool	1 × 1 × 64
Max Pooling	-
SoftMax	classifier

**Table 4:** Structure of Tiny MobileNet V2: each line describes a sequence that repeats the same layer *n* times. All layers in the same sequence have the same number *c* of output channels. The module repeats stride *s* for the first time, and all others use stride 1. The expansion factor *t* is always applied to the input size.

Operator	t	c	n	s
Conv2d 3 × 3	-	32	1	2
Bottleneck	1	16	1	2
Bottleneck	6	24	2	2
Conv2d 1 × 1	-	64	1	2
MaxPool 7 × 1	-	-	1	-
Conv2d 1 × 1	-	3	-	-

**Table 5:** Results of the three outline-based convolutional networks: different actions of wild felines produce different results for different networks.

	Tiny MobileNet V2	Tiny Inception V3	Tiny VGG
**Galloping**	79%	77%	96%
**Standing**	88%	89%	95%
**Ambling**	82%	74%	84%
**Average accuracy**	83%	80%	92%

**Table 6:** Accuracy (%) of the ablation study: the outline-only method applies Outline Mask RCNN for target feline outline and Tiny VGG for action recognition; the skeleton-only method use LEAP to obtain target feline skeleton and Long Short-Term Memory (LSTM) for action recognition; and the two-stream method incorporates the above two methods for action recognition.

	Galloping	Standing	Ambling	Average Accuracy
Outline-only method	96%	95%	84%	92%
Skeleton-only method	83%	93%	91%	89%
Two-stream method	97%	97%	93%	95%

## Data Availability

The data presented in this study are available on request from the corresponding author.

## References

[1] Fraser A.F., Hulbert S., Lainsbury A., Head T. (2012). Introduction. Feline Behaviour and Welfare.

[2] Atkinson T., Makepeace C., Lainsbury A., Kapp T. (2018). The Origin and Evolution of the Domestic Cat. Practical Feline Behaviour Understanding Cat Behaviour and Improving Welfare.

[3] Marchant-Forde J.N. (2015). The science of animal behavior and welfare: Challenges, opportunities and global perspective. Front. Vet. Sci..

[4] Anderson D.J., Perona P. (2014). Toward a science of computational ethology. Neuron.

[5] Biolatti C., Modesto P., Dezzutto D., Pera F., Tarantola M., Gennero M.S., Maurella C., Acutis P.L. (2016). Behavioural analysis of captive tigers Pantheratigris: A water pool makes the difference. Appl. Anim. Behav. Sci..

[6] Shepherdson D.J., Mellen J.D. (1998). Second Nature Environmental Enrichment for Captive Animals.

[7] Vaz J., Narayan E.J., Dileep Kumar R., Thenmozhi K., Thiyagesan K., Baskaran N. (2017). Prevalence and determinants of stereotypic behaviours and physiological stress among tigers and leopards in Indian zoos. PLoS ONE.

[8] Chakravarty P., Maalberg M., Cozzi G., Ozgul A., Aminian K. (2019). Behavioural compass: Animal behaviour recognition using magnetometers. Mov. Ecol..

[9] Williams H.J., Holton M.D., Shepard E.L., Largey N., Norman B., Ryan P.G., Duriez O., Scantlebury M., Quintana F., Magowan E.A. (2017). Identification of animal movement patterns using tri-axial magnetometry. Mov. Ecol..

[10] Noda T., Kawabata Y., Arai N., Mitamura H., Watanabe S. (2014). Animal-mounted gyroscope/ accelerometer/ magnetometer: In situ measurement of the movement performance of fast-start behaviour in fish. J. Exp. Mar. Biol. Ecol..

[11] Mench J.A. (1998). Why it is important to understand animal behavior. ILAR J..

[12] Li Q., Yuan P., Liu X., Zhou H. (2020). Street tree segmentation from mobile laser scanning data. Int. J. Remote Sens..

[13] Akçay H.G., Kabasakal B., Aksu D., Demir N., Öz M., Erdoğan A. (2020). Automated Bird Counting with Deep Learning for Regional Bird Distribution Mapping. Animals.

[14] Agbele T., Ojeme B., Jiang R. (2019). Application of local binary patterns and cascade AdaBoost classifier for mice behavioural patterns detection and analysis. Proced. Comput. Sci..

[15] Jiang Z., Crookes D., Green B.D., Zhang S., Zhou H. (2017). Behaviour recognition in mouse videos using contextual features encoded by spatial-temporal stacked Fisher vectors. ICPRAM.

[16] Nguyen N., Delimayanti M., Purnama B., Mahmudah K., Kubo M., Kakikawa M., Yamada Y., Satou K. (2019). Applying Deep Learning Models to Action Recognition of Swimming Mice with the Scarcity of Training Data. Bioinformatics.

[17] Lorbach M., Poppe R., Veltkamp R.C. (2019). Interactive rodent behavior annotation in video using active learning. Multimed. Tools Appl..

[18] Gu J.Q., Wang Z.H., Gao R.H., Wu H.R. (2017). Cow behavior recognition based on image analysis and activities. Int. J. Agric. Biol. Eng..

[19] He D.J., Meng F.C., Zhao K.X., Zhang Z. (2016). Recognition of Calf Basic Behaviors Based on Video Analysis. Trans. CSAM.

[20] Li J. (2018). Study on Identification of Typical Cow‘s Self-Protective Behavior Based on Machine Vision Technology. Ph.D. Thesis.

[21] Lee J., Jin L., Park D., Chung Y. (2016). Automatic Recognition of Aggressive Behavior in Pigs Using a Kinect Depth Sensor. Sensors.

[22] Luo Y., Wang L., Yang L., Tan M., Wu Y., Li Y., Li Z. (2018). Puppet resting behavior in the Tibetan antelope (Pantholops hodgsonii). PLoS ONE.

[23] Bod‘ová K., Mitchell G.J., Harpaz R., Schneidman E., Tkačik G. (2018). Probabilistic models of individual and collective animal behavior. PLoS ONE.

[24] George G., Namdev A., Sarma S. (2018). Animal Action Recognition: Analysis of Various Approaches. Int. J. Eng. Sci. Res. Technol..

[25] Pereira T.D., Aldarondo D.E., Willmore L., Kislin M., Wang S.S.H., Murthy M., Shaevitz J.W. (2019). Fast animal pose estimation using deep neural networks. Nat. Methods.

[26] Simonyan K., Zisserman A. (2014). Very deep convolutional networks for large-scale image recognition. Comput. Sci..

[27] Jaouedi N., Perales F.J., Buades J.M., Boujnah N., Bouhlel M.S. (2020). Prediction of Human Activities Based on a New Structure of Skeleton Features and Deep Learning Model. Sensors.

[28] Lin T., Zhao X., Su H., Wang C.J., Yang M. (2018). BSN: Boundary Sensitive Network for Temporal Action Proposal Generation. Proceed. Eur. Conf. Comput. Vis. ECCV.

[29] Okafor E., Pawara P., Karaaba F., Surinta O., Codreanu V., Schomaker L., Wiering M. Comparative study between deep learning and bag of visual words for wild animal recognition. Proceedings of the 2016 IEEE Symposium Series on Computational Intelligence (SSCI).

[30] Feichtenhofer C., Pinz A., Zisserman A. (2016). Convolutional two-stream network fusion for video action recognition. Proceed. IEEE Conf. Comput. Vis. Pattern Recognit..

[31] Gómez A., Salazar A., Vargas F. (2017). Towards Automatic Wild Animal Monitoring: Identification of Animal Species in Camera-trap Images using Very Deep Convolutional Neural Networks. Ecol. Inform..

[32] Falzon G., Lawson C., Cheung K.-W., Vernes K., Ballard G.A., Fleming P.J.S., Glen A.S., Milne H., Mather-Zardain A., Meek P.D. (2020). ClassifyMe: A Field-Scouting Software for the Identification of Wildlife in Camera Trap Images. Animals.

[33] Chen G., Han T.X., He Z., Kays R., Forrester T. Deep convolutional neural network based species recognition for wild animal monitoring. Proceedings of the 2014 IEEE International Conference on Image Processing (ICIP).

[34] Norouzzadeha M.S., Nguyenb A., Kosmalac M., Swansond A., Palmer M.S., Packer C., Clune J. (2018). Automatically identifying, counting, and describing wild animals in camera-trap images with deep learning. Proc. Nat. Acad. Sci. USA.

[35] Zhang T., Liu L., Zhao K., Wiliem A., Hemson G., Lovell B. (2018). Omni-supervised joint detection and pose estimation for wild animals. Pattern Recognit. Lett..

[36] Wark J.D., Cronin K.A., Niemann T., Shender M.A., Horrigan A., Kao A., Ross M.R. (2019). Monitoring the behavior and habitat use of animals to enhance welfare using the ZooMonitor app. Anim. Behav. Cognit..

[37] Zuffi S., Kanazawa A., Jacobs D., Black M.J. (2017). 3D Menagerie. Modeling the3D Shape and Pose of Animals. Comput. Vis. Pattern Recognit. Int. Conf..

[38] Romero-Ferrero F., Bergomi M.G., Hinz R.C., Heras F.J., De Polavieja G.G. (2019). Idtracker. ai: Tracking all individuals in small or large collectives of unmarked animals. Nat. Methods.

[39] He K., Gkioxari G., Dollár P., Girshick R. (2017). Mask R-CNN. Proceed. IEEE Int. Conf. Comput. Vis..

[40] Girshick R. (2015). Fast r-cnn. Proceed. IEEE Int. Conf. Comput. Vis..

[41] Long J., Shelhamer E., Darrell T. (2015). Fully convolutional networks for semantic segmentation. Proceed. IEEE Conf. Comput. Vis. Pattern Recognit..

[42] Zhang Y., Tian Z., Lei Y., Wang T., Patel P., Jani A.B., Curran W.J., Liu T., Yang X. (2020). Automatic multi-needle localization in ultrasound images using large margin mask RCNN for ultrasound-guided prostate brachytherapy. Phys. Med. Biol..

[43] Tao C., Jin Y., Cao F., Zhang Z., Li C., Gao H. (2020). 3D Semantic VSLAM of Indoor Environment Based on Mask Scoring RCNN. Discrete Dyn. Nat. Soc..

[44] Rohit Malhotra K., Davoudi A., Siegel S., Bihorac A., Rashidi P. (2018). Autonomous detection of disruptions in the intensive care unit using deep mask RCNN. Proceed. IEEE Conf. Comput. Vis. Pattern Recognit. Workshops.

[45] Swanson A., Kosmala M., Lintott C., Simpson R., Smith A., Packer C. (2015). Snapshot Serengeti, high-frequency annotated camera trap images of 40 mammalian species in an African savanna. Sci. Data.

[46] Lin T.Y., Maire M., Belongie S., Hays J., Perona P., Ramanan D., Dollar P., Zitnick C.L. (2014). Microsoft coco: Common objects in context. European Conference on Computer Vision.

[47] He K., Zhang X., Ren S., Sun J. (2016). Deep residual learning for image recognition. Proceed. IEEE Conf. Comput. Vis. Pattern Recognit..

[48] Bridle J.S. (1990). Probabilistic interpretation of feedforward classification network outputs, with relationships to statistical pattern recognition. Neurocomputing.

[49] Olshausen B.A., Field D.J. (1996). Emergence of simple-cell receptive field properties by learning a sparse code for natural images. Nature.

[50] Glorot X., Bordes A., Bengio Y. Deep sparse rectifier neural networks. Proceedings of the 14th International Conference on Artificial Intelligence and Statistics.

[51] Zhao F., Hung D.L., Wu S. (2020). K-means clustering-driven detection of time-resolved vortex patterns and cyclic variations inside a direct injection engine. Appl. Therm. Eng..

[52] Greff K., Srivastava R.K., Koutník J., Steunebrink B.R., Schmidhuber J. (2017). LSTM: A Search Space Odyssey. IEEE Trans. Neural Networks Learn. Syst..

[53] Zhang H., Zhang J., Shi K., Wang H. (2020). Applying Software Metrics to RNN for Early Reliability Evaluation. J. Control Sci. Eng..

[54] Python: An All-in-One Web Crawler, Web Parser and Web Scrapping Library!. https://psgithub.com/hardikvasa/webb.

[55] Dangtongdee K.D. (2018). Plant Identification Using Tensorflow.

[56] Kingma D.P., Ba J. (2014). Adam: A Method for Stochastic Optimization.

[57] Sandler M., Howard A., Zhu M., Zhmoginov A., Chen L.C. (2018). MobileNetV2: Inverted Residuals and Linear Bottlenecks. Proceed. IEEE Conf. Comput. Vis. Pattern Recognit..

[58] Szegedy C., Vanhoucke V., Ioffe S., Shlens J., Wojna Z. (2016). Rethinking the inception architecture for computer vision. Proceed. IEEE Conf. Comput. Vis. Pattern Recognit..

[59] Nath T., Mathis A., Chen A.C., Patel A., Bethge M., Mathis M.W. (2019). Using DeepLabCut for 3D markerless pose estimation across species and behaviors. Nat. Protoc..

[60] Graving J.M., Chae D., Naik H., Li L., Koger B., Costelloe B.R., Couzin I.D. (2019). DeepPoseKit, a software toolkit for fast and robust animal pose estimation using deep learning. Elife.

[61] Cui Z. (2020). On the Cover: Violin Plot. Educ. Meas. Issues Pract..

[62] Ndako J.A., Olisa J.A., Ifeanyichukwu I.C., Ojo S.K.S., Okolie C.E. (2020). Evaluation of diagnostic assay of patients with enteric fever by the box-plot distribution method. N. Microbes N. Infect..

[63] Cao Z., Simon T., Wei S.E., Sheikh Y. (2017). Realtime multi-person 2d pose estimation using part affinity fields. Proceed. IEEE Conf. Comput. Vis. Pattern Recognit..

[64] Yun K., Honorio J., Chattopadhyay D., Berg T.L., Samaras D. Two-person Interaction Detection Using Body-Pose Features and Multiple Instance Learning. Proceedings of the 2012 IEEE Computer Society Conference on Computer Vision and Pattern Recognition Workshops.

[65] Yan S., Xiong Y., Lin D. (2018). Spatial temporal graph convolution for skeleton-based action recognition. Proceedings of the AAAI Conference on Artificial Intelligence.

[66] Si C., Chen W., Wang W., Wang L., Tan T. (2019). An attention enhanced graph convolutional lstm network for skeleton-based action recognition. Proceed. IEEE Conf. Comput. Vis. Pattern Recognit..

